# *Salmonella*-induced pulmonary and pericardial abscesses in a patient presenting with subacute cough

**DOI:** 10.1016/j.idcr.2022.e01430

**Published:** 2022-01-28

**Authors:** S. Ismail, M. Thomas, Almurtada Razok, R. Akbar, Fatma Ben Abid, Godwin Wilson

**Affiliations:** a Department of Chest, Hamad General Hospital, P.O. Box 3050, Doha, Qatar; b Department of Medicine, Weill Cornell Medical College, Qatar; c Department of Internal Medicine, Hamad General Hospital, P.O. Box 3050, Doha, Qatar; d Department of Infectious diseases, Communicable Diseases Centre, Hamad Medical Corporation, P.O. Box 3050, Doha, Qatar; e Department of Microbiology, Hamad General Hospital, P.O. Box 3050, Doha, Qatar

**Keywords:** *Salmonella*, Pulmonary abscess, Pericardial abscess, Bronchoscopy

## Abstract

The non-typhoid *Salmonella* (NTS) species are commonly associated with gastroenteritis and other forms of intestinal disease. Thoraco-pulmonary infections are less commonly reported. We describe the case of a 66-year-old Qatari lady who presented with subacute cough. Chest imaging revealed multiple pulmonary and a pericardial cavitary lesion with air fluid levels. Bronchoalveolar lavage culture grew *Salmonella* species group D. The patient was treated with 4 weeks of appropriate antibiotics. Clinical and radiological improvement were documented on subsequent follow up. To our knowledge, this is the first reported case of pulmonary and pericardial *salmonella* abscesses in the state of Qatar.

## Introduction

*Salmonellae* are gram-negative *Enterobacteriaceae* which commonly cause gastroenteritis. Enteric fever is caused by *Salmonella typhi* and Salmonella paratyphi. Other serotypes of *Salmonella* are commonly referred to as non-typhoidal *salmonella* (NTS) [1]. *Salmonellosis* is usually caused by ingestion of poultry, eggs, and milk products, however NTS have been associated with contact to animals, including reptiles [2]. Invasive infections due to NTS occur globally, but the burden varies by geographic location. Approximately 1% of enteric infections with NTS result in bacteremia [3].

NTS bacteremia can disseminate to any site, including the genitourinary, thoraco-pulmonary, cardiovascular, musculoskeletal, and central nervous systems. Extraintestinal manifestations of NTS infection are rare and usually occur in patients with certain risk factors like extremes of age, immunocompromising conditions, chronic liver disease, chronic granulomatous diseases, HIV (human immunodeficiency virus) and hemoglobinopathies (especially sickle cell disease) [4]. In specific, the pleuro-pulmonary manifestations are quite uncommon and occur primarily in patients with pre-existing lung disease [5]. We report here a case of an elderly patient with multiple lung and paracardiac abscesses caused by NTS.

## Case presentation

**Fig. 1:**
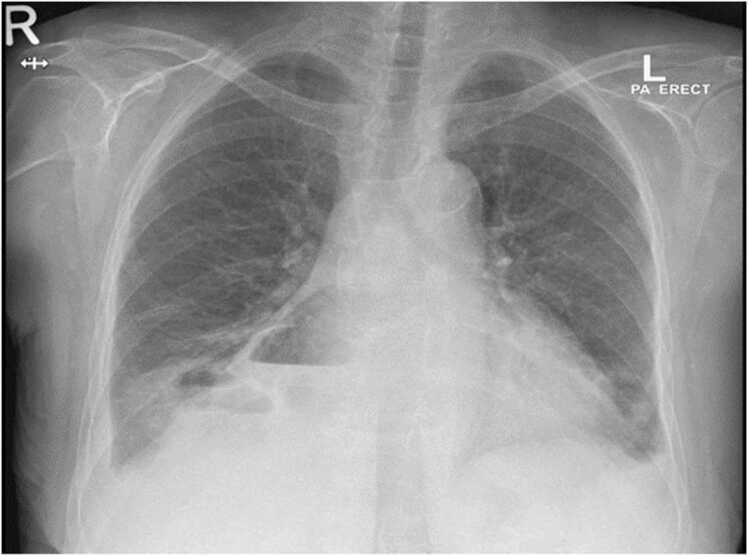
Posteroanterior chest Xray showing cavitary lesions with multiple air fluid levels silhouetting the right heart border and right hemidiaphragm accompanied by a mild right-sided pleural effusion.

**Fig. 2:**
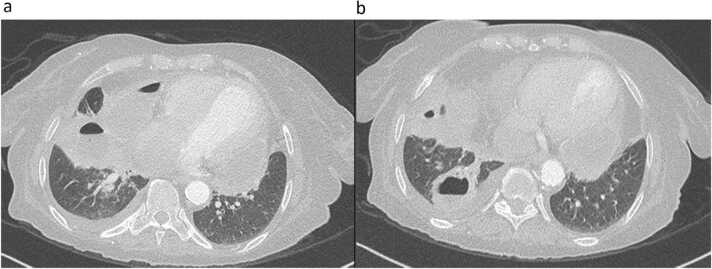
Computed tomography scan of the chest showing multiple cavitary lesions in the right middle and lower lobes of the lung, the largest of which measuring 5.5 × 4.2 × 3.5 cm (a) and a similar right pericardial lesion along the right heart border measuring around 8.5 × 6.2 × 3.6 cm (b).

**Fig. 3:**
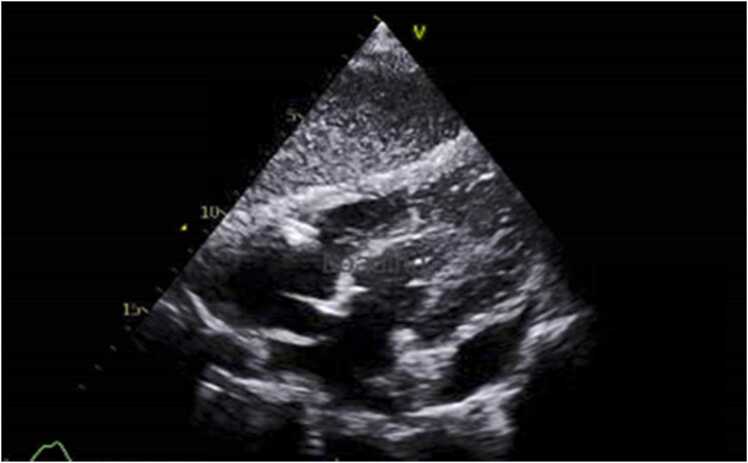
Echocardiogram showing loculated pericardial effusion measuring around 2 cm with no signs of tamponade.

A 66-year-old lady with background of diabetes mellitus, hypertension, and end-stage renal disease on regular hemodialysis, presented to our hospital with productive cough of four weeks duration. There was no associated fever, chest pain, dyspnea, night sweats or weight loss. She did not report any recent or remote gastrointestinal symptoms. There was no history of exposure to sick contacts or animals. On examination, she was comfortable at rest and afebrile with normal vital signs. Respiratory examination revealed decreased breath sounds with dullness to percussion over the right infra-scapular area. Blood tests revealed a white blood cell count of 12 × 10^3^/uL (reference range 4–10 × 10^3^/uL) with 75% neutrophils and elevated C-reactive protein of 137 mg/L (reference range 0–5 mg/L). Stool culture and ova/parasite detection were negative. Chest Xray showed cavitary leisons with multiple air fluid levels silhouetting the right heart border and right hemidiaphragm with mild right-sided pleural effusion (Fig. 1). Computed tomography (CT) scan of the chest showed multiple cavitary lesions in the right middle and lower lobes of the lung, the largest measuring 5.5 × 4.2 × 3.5 cm, and a similar right pericardial lesion along the right heart border measuring 8.5 × 6.2 × 3.6 cm (Fig. 2a, b). Echocardiogram showed a loculated pericardial effusion, adjacent to the lateral left ventricle measuring 2 cm with no signs of cardiac tamponade (Fig. 3). Sputum and blood cultures were negative. The patient was empirically started on piperacillin/tazobactam. Sputum smear, polymerase chain reaction (PCR) and culture from both sputum and BAL for Mycobacteria were also negative. Culture of bronchoalveolar lavage fluid (BAL) grew non lactose fermenting gram negative bacilli that was identified as *Salmonella* Group D, sensitive to ceftriaxone. Based on the above, antibiotic was deescalated to ceftriaxone 2 gm daily.

The patient remained afebrile and vitally stable throughout her stay in the hospital. She was discharged home on day six and received intravenous ceftriaxone for two weeks at home followed by two weeks of oral cefuroxime. She was seen in the pulmonology clinic six weeks after discharge, and she reported improvement in her cough. Repeat chest Xray eight weeks after discharge showed significant regression in the cavitary lesions and associated air-fluid levels. In addition, a transthoracic echocardiogram performed four months after the initial presentation showed complete resolution of the pericardial abscess and no evidence of pericardial effusion. CT chest was performed six months after the initial presentation and showed complete resolution of the previously detected pulmonary cavitary lesions.

## Discussion

The prevalence of invasive infections caused by NTS is increasing worldwide among immunocompromised patients, particularly in developing countries, however they are quite uncommon in immunocompetent hosts [6]. Intrathoracic infection as an extra-intestinal manifestation is very rare with only few cases reported in the literature so far [7]. The risk factors that can lead to intrathoracic NTS infections can be broadly categorized into systemic and local. Systemic factors include HIV, malaria, diabetes mellitus, uremia, impaired cell-mediated immunity, impaired B-cell function, prior use of antibiotics, diminished gastric acidity, and low socioeconomic status. Local factors include prior lung or pleural disease and congenital abnormalities [8], [9], [10]. Our patient had multiple comorbidities including end stage renal disease (ESRD) requiring hemodialysis. ESRD is not categorically labeled as an immunocompromised state [11]. However, both innate and adaptive immunity are deranged from the inflammatory uremic milieu in patients with decreased renal function [12]. The killing capability of neutrophils is reduced in end-stage renal disease (ESRD) patients and the functional impairment described in uremic neutrophils is therefore mainly a result of their reduced ability to kill microorganisms intracellularly and is believed to increase the susceptibility to infections. Inflammations secondary to Hemodialysis further adds to the altered immune system [13]. These immune system disturbances could explain why our patient developed NTS lung abscess in the absence of traditional risk factors known to be associated with extraintestinal disease associated with salmonellosis.

Systemic dissemination of the organism is most likely to occur through hematogenous spread via the reticuloendothelial system. Hematogenous spread can be transient leading to negative blood cultures such as our patient [14]. *Salmonella* was isolated from BAL and the patient showed clinical and radiological improvement with appropriate antibiotics which she received for six weeks in total.

The duration of antibiotic treatment for disseminated salmonella lung abscesses is not well defined in literature. The choice and duration of antibiotics usually depend on the susceptibility of the organism and the host response. Most of the cases improve with antibiotics alone. Some cases may require surgical intervention for clearance of infection, if not responding to antibiotics or when the focus of infection cannot be penetrated by antimicrobials [10]. However, our patient showed good response to antibiotics alone.

In the following table we review some of the previously reported cases of *Salmonella* lung abscesses and empyema in patients without pre-existing lung disease (Table 1). Vassiliki Pitiriga et al. reported a case of right sided pulmonary abscess in an immunocompetent host in 2016, however the patient required lobectomy in addition to antimicrobial therapy to achieve clinical improvement [10]. Rim et al. and Ramanathan et al. reported cases of empyema and both patients improved with antibiotics and repeated pleural drainage [15], [16].

**Table 1:** Review of reported cases of pulmonary infections caused by *salmonella* in patients without pre-existing lung disease.

Author	Age	Comorbid conditions	Pulmonary manifestation	Treatment	Outcome
Pitiriga et al. [10]	26 years	None	Right sided lung abscess	Antibiotics + Lobectomy	Improved
Rim et al. [15]	70 years	Diabetes mellitus	Empyema	Antimicrobial therapy and repeated therapeutic thoracentesis	Improved
Ramanathan et al*.*[16]	50 years	Diabetes mellitus and gallbladder carcinoma	Left Pleural Empyema	Antibiotic treatment + Intercostal tube drainage	Improved
Gopinath et al. [17]	18 years	None	Rt. Pleural Empyema	Chloramphenicol open drainage, thoracoplasty	Improved
Rao and Sattar [18]	30 years	None	Lt. Pleural effusion	Chloramphenicol + Closed thoracic drainage	Improved
Annamalai et al. [19]	47 years	None	Rt. Pleural Empyema	Chloramphenicol + thoracocentesis	Improved
Nair et al. [20]	Adult	Esophageal stricture	left-sided pneumonia	Antibiotics	Improved
Gupta et al. [21]	Adult	None	Left sided lung abscess Salmonella Group E isolated from stool culture	Antibiotics	Improved

## Conclusion

Intrathoracic salmonellosis can occur in patients with or without pre-existing lung disease. Timely initiation of appropriate antibiotics can reduce the morbidity and mortality and can obviate the need for surgical interventions. This case raises the awareness of Group D Salmonella as a potential pathogen for lung and pericardial abscesses in an immunocompetent patient. Early diagnosis and appropriate antibiotic treatment can spare the patient from invasive surgery.

## Funding

This article did not receive any specific grant from funding agencies in the public, commercial, or not-for-profit sectors.

## Ethical approval

Yes.

## Consent

Written informed consent was obtained from the patient for publication of this case report and the accompanying images. A copy of the written consent is available for review by the Editor-in-Chief of this journal on request.

## CRediT authorship contribution statement

**S. Ismail:** Performed literature review and Writing – original draft of the manuscript. **Almurtada Razok:** Performed literature review and Writing – original draft of the manuscript. **M. Thomas:** Supervised the writing process and revised the manuscript. **R. Akbar:** Supervised the writing process and revised the manuscript. **Fatma Ben Abid:** Supervised the writing process and revised the manuscript. **Godwin Wilson:** Supervised the writing process and revised the manuscript. All authors approved the final version for submission.

## Competing interests

None to be declared.

## Data Availability

The datasets used and/or analyzed during the current study are available from the corresponding author on reasonable request.
